# Effects of Exercise Addiction and the *COL1A1* Gene rs1800012 Polymorphism on Injury Susceptibility in Elite Female Volleyball Players

**DOI:** 10.3390/genes16111300

**Published:** 2025-11-01

**Authors:** Muhammed Mustafa Piri, Mesut Cerit, Murat Anılır, Tolga Polat, Aynur Ayşe Karaduman, Attila Szabo, Tiffany Georges Abi Antoun, George John, Ekaterina A. Semenova, Andrey K. Larin, Nikolay A. Kulemin, Edward V. Generozov, Ildus I. Ahmetov

**Affiliations:** 1 Department of Exercise and Sports Science, Lokman Hekim University, 06510 Ankara, Türkiye; 2 Department of Sports Management, Lokman Hekim University, 06510 Ankara, Türkiye; 3 Faculty of Dentistry, Basic Medical Sciences, Department of Basic Health Sciences, Marmara University, 34722 Istanbul, Türkiye; 4 Faculty of Health Sciences, Department of Physiotherapy, Lokman Hekim University, 06510 Ankara, Türkiye; 5 Faculty of Health and Sport Sciences, Széchenyi István University, 9026 Győr, Hungary; 6 School of Medicine and Medical Sciences, Holy Spirit University of Kaslik, Jounieh P.O. Box 446, Lebanon; 7 Transform Specialist Medical Centre, Dubai 119190, United Arab Emirates; 8 Department of Molecular Biology and Genetics, Lopukhin Federal Research and Clinical Center of Physical-Chemical Medicine of Federal Medical Biological Agency, 119435 Moscow, Russia; 9 Research Institute of Physical Culture and Sport, Volga Region State University of Physical Culture, Sport and Tourism, 420138 Kazan, Russia; 10 Laboratory of Genetics of Aging and Longevity, Kazan State Medical University, 420111 Kazan, Russia; 11 Research Institute for Sport and Exercise Sciences, Liverpool John Moores University, Liverpool L3 5AF, UK

**Keywords:** polymorphism, *COL1A1*, volleyball, exercise addiction, sport injuries

## Abstract

Objectives: The objective of this study was to separately examine the effects of exercise addiction and the Collagen Type I Alpha 1 Chain (*COL1A1*) gene rs1800012 G/T polymorphism on injury susceptibility in elite female volleyball players, and to test the hypothesis that the T allele, previously identified as a risk allele, is underrepresented in volleyball players compared to the general population. Methods: The study included 50 professional Turkish female volleyball players with documented injury data, along with 557 Turkish controls, 53 professional Russian volleyball players, and 810 Russian controls. The Turkish participants were enrolled in a case–control study, an injury study, and an exercise addiction study, whereas the Russian participants were enrolled solely in a case–control study. Results: Injured players had significantly higher scores in the Delay of Individual Social Needs and Conflict subscale of the Exercise Addiction Scale compared to their uninjured counterparts (*p* = 0.036). The random-effects meta-analysis revealed a significantly lower frequency of the *COL1A1* T allele in volleyball players compared to controls (pooled OR = 0.63, 95% CI: 0.41–0.96, *p* = 0.031). Athletes who had not undergone surgery had a significantly higher frequency of the G allele compared to controls (89.2% vs. 78.7%, *p* = 0.037; OR = 2.23, 95% CI: 1.1–4.7). Among injured athletes, those carrying the GT genotype were significantly more likely to experience prolonged recovery (≥3 months) (57.1%) compared to those with the GG genotype (28.0%, *p* = 0.017). Conclusions: Exercise addiction and the *COL1A1* rs1800012 T allele were associated with a higher incidence of injury in female volleyball players. The T allele was also associated with a longer recovery time following injury.

## 1. Introduction

Injuries significantly impact the careers of athletes and have long-term consequences for their health. Recurrent musculoskeletal injuries, such as ligament tears, stress fractures, and muscle strains, can lead to post-traumatic osteoarthritis and chronic pain [1,2]. For instance, the study by Lohmander et al. [3] reported a remarkably high prevalence of knee-related issues among young female soccer players who had sustained an anterior cruciate ligament (ACL) tear 12 years earlier. The findings revealed that 82% of participants exhibited radiographic changes in the affected knee, 51% met the criteria for radiographic knee osteoarthritis (OA), and 75% reported symptoms that negatively impacted their knee-related quality of life [3]. Additionally, the psychological impact of injuries, including depression, anxiety, and fear of re-injury, can hinder an athlete’s ability to return to peak performance and may contribute to early retirement from sports [4,5]. Preventative measures, such as strength training, proper warm-up routines, and injury surveillance programs, have been shown to reduce injury rates by up to 50% in some sports [6,7]. Despite these advancements, the long-term health consequences of sports injuries remain a critical concern, particularly for athletes who sustain multiple injuries over their careers. This underscores the need for continued research into injury prevention, rehabilitation strategies, and long-term health monitoring for athletes [8].

Several factors contribute to injury susceptibility, including genetic, hormonal (sex-specific), biomechanical, environmental, and physiological aspects [9]. Training load, recovery periods, biomechanics, and neuromuscular control play crucial roles in injury risk [10,11,12]. Psychological factors, such as stress and mental fatigue, further influence injury occurrence by impairing concentration and motor control [13].

Exercise addiction is an emerging psychological factor that exacerbates injury risk. It is characterized by compulsive physical activity despite adverse consequences, often accompanied by withdrawal symptoms when exercise is reduced [14]. Studies indicate that athletes possess a greater susceptibility to exercise addiction compared to non-athletes [15]. Athletes with exercise addiction tend to engage in excessive training volumes, leading to overuse injuries, stress fractures, and immune suppression, which may impair recovery [14,16]. The psychological drive behind excessive training often leads to neglecting injury symptoms, resulting in chronic damage and prolonged rehabilitation periods [17].

Genetic variants contribute to individual differences in injury susceptibility [18,19,20], as well as to exercise addiction [21], physical activity [22], and athletic performance [23,24,25,26]. The concept of “elite athlete” status often involves a selection process where genetic profiles that confer resilience or a lower risk of injury may be enriched. Numerous single-nucleotide polymorphisms (SNPs) have been identified as associated with musculoskeletal injuries [27,28,29,30,31]. Soft tissues, particularly collagens that connect ligaments, tendons, and muscles, influence joint mobility and injury risk. Type I collagen, the predominant form in the human body, is essential for tendon and ligament integrity and is encoded by the *COL1A1* gene (which encodes the collagen type I alpha 1 chain) [18,19]. A G>T transversion (rs1800012) at the Sp1 transcription factor binding site within the first intron of this gene affects the production of type I collagen fibers, resulting in weaker fibers [32,33]. According to the GTEx portal (https://gtexportal.org; accessed on 24 August 2025), individuals with the less frequent TT genotype exhibit the lowest *COL1A1* expression in skeletal muscle (*p* = 0.0458) and leg skin (*p* = 0.0151) compared to other genotypes, suggesting increased vulnerability to injuries. Indeed, two studies have reported that the T allele may be a risk factor for soft tissue injuries in both athletes and physically active subjects [34,35]. Other rare and common variants in the *COL1A1* gene have also been associated with flexibility and joint mobility [36,37].

Given that the T allele is associated with weaker collagen fibers and increased injury risk, we hypothesize that it may be negatively selected in elite sports populations. In other words, individuals carrying this allele may be less likely to reach the elite level due to a higher propensity for injury, which would be a significant disadvantage in a high-impact sport like volleyball. Understanding the interplay between exercise addiction and genetic predispositions is essential for developing personalized injury prevention strategies.

Therefore, the primary aim of this study was to separately investigate the associations of (1) exercise addiction and (2) the *COL1A1* rs1800012 G/T polymorphism with injury susceptibility in elite female volleyball players. Additionally, based on the established role of the T allele as a risk factor for soft-tissue injuries, we proposed that it might be negatively selected in elite sports. Consequently, a secondary aim was to test the specific hypothesis that the T allele is underrepresented in elite volleyball players compared to the general population.

## 2. Materials and Methods

### 2.1. Ethics Statement

The study was conducted following the Declaration of Helsinki, and ethical approval was obtained from the Lokman Hekim University Non-Interventional Clinical Research Ethics Committee (reference 2025-110; approval date: 28 April 2025) and the Ethics Committee of the Federal Research and Clinical Center of Physical–Chemical Medicine of the Federal Medical and Biological Agency of Russia (reference 2017/04; approval date: 4 July 2017). Informed consent was obtained from all participants.

### 2.2. Participants

The study included 1470 Turkish and Russian subjects of European descent, comprising 103 professional volleyball players and 1367 controls. Turkish participants were enrolled in a case–control study, an injury study, and an exercise addiction study, whereas Russian participants were enrolled solely in a case–control study.

Among the 103 volleyball players, 50 were professional Turkish female athletes (mean age: 26.2 ± 4.7 years) representing five clubs from the national professional league, for whom injury data were available. To be eligible, the Turkish players had to have competed in the professional league for at least four years and no more than twelve years. Throughout the volleyball season, the athletes took part in one official match each week and completed six training sessions, each lasting around 45–75 min, which emphasized technical skills, tactical strategies, and physical conditioning. Their characteristics are presented in Table 1. No control group was used for this injury analysis; comparisons were made within this cohort based on genotype and behavioral data. For the case–control study, *COL1A1* rs1800012 genotype data from 557 healthy individuals serving as controls (non-athletes) were obtained from the Turkish Genome Project (https://tgd.tuseb.gov.tr/en/; accessed on 24 August 2025). The control participants had no prior experience in elite sports. Although specific data on recreational physical activity or training volume were not collected, the cohort reflects the general population and adhered to strict inclusion criteria that excluded individuals with chronic or hereditary conditions. All Turkish athletes and control subjects were of Caucasian ancestry.

The Russian athlete cohort comprised 53 professional volleyball players (14 females, mean age: 22.7 ± 0.5 years; 39 males, mean age: 26.1 ± 3.9 years). Among them, 22 were classified as highly elite, having won prizes in international competitions such as the Olympic Games, World and European Championships; 17 were considered elite, with prizes in national competitions; and 14 were sub-elite, without national-level awards. Muscle injury data were not available for these athletes, as this cohort was included solely to assess *COL1A1* rs1800012 allele frequencies in comparison with controls. The Russian control group comprised 810 individuals, including 640 healthy donors and patients with multifactorial conditions, as previously described by Barbitoff et al. [38], along with an additional 170 controls (mean age: 44.5 ± 4.1 years; 47 females, 123 males) who had no prior experience in elite sports. Detailed information regarding physical activity or sports participation was not recorded for these controls. All Russian athletes and controls were of Caucasian ancestry.

### 2.3. Experimental Approach

The Turkish participants were informed about the study and measurement protocols one week in advance, and consent forms were signed on the day of measurement. Demographic data, including age, height, weight, playing position, collagen supplementation, history of injuries with medical documentation of surgical procedures, and post-injury recovery duration, were collected through structured inquiries. All injuries were corroborated by official medical records, and only recorded cases were incorporated into the study. Diagnoses were determined by team physicians using standardized clinical examinations and, when required, corroborated by imaging techniques such as magnetic resonance imaging (MRI) or ultrasound.

### 2.4. Genotyping

Oral swab samples for DNA analysis were obtained from Turkish volleyball players by appointment before training sessions during the season using the buccal swab technique and subsequently stored at +4 °C. Genomic DNA was extracted using the ZipPrime Epithelial Cell DNA Extraction Kit (ZipPrime, Ankara, Türkiye), which was designed for high-quality DNA extraction from oral epithelial cells. The obtained genomic DNA was analyzed using the ZipPrime Life Style Real-Time PCR Kit, and the rs1800012 SNP was identified through Fluorescence Melting Curve Analysis (FMCA). Genomic DNA extraction and *COL1A1* rs1800012 SNP analysis were performed using the SLAN 96-P Real-Time PCR system in conjunction with the Life Style Real-Time PCR Kit (Athletic Performance Module, Ankara, Türkiye). The PCR reaction mixture consisted of 12.5 µL of 2× Mastermix, 5 µL of 5× Primer Mix (containing *COL1A1* forward and reverse primers with FAM-HEX probes), 2.5 µL of DNase/RNase-free dH2O, and 5 µL of template DNA.

In the Russian cohort, DNA was isolated from leukocytes obtained from 4 mL of venous blood from 53 athletes and 170 controls. Extraction and purification were performed with a commercial kit according to the manufacturer’s instructions (Technoclon, Moscow, Russia). Genotyping of the *COL1A1* rs1800012 variant was conducted using Illumina microarray platforms (HumanOmni1-Quad and HumanOmniExpress BeadChips; Illumina, San Diego, CA, USA), as previously reported [26]. For the additional Russian control group (n = 640), genotype information was derived from whole-exome sequencing, as described earlier [38], with the data available through the RUSeq database [39].

### 2.5. Evaluation of Exercise Addiction

Exercise addiction was assessed using the Exercise Addiction Scale (EAS) developed by Demir et al. [40]. This scale comprises three subscales: “Excessive Focus and Emotional Change,” “Delaying Personal and Social Needs and Conflict,” and “Tolerance Development and Passion.” The participants were classified into five categories based on their exercise addiction scores. The scale ratings were as follows: 1 = Strongly Disagree, 2 = Partially Disagree, 3 = Moderately Agree, 4 = Agree, and 5 = Strongly Agree. Score classifications were defined as follows: 1–17 (normal group), 18–34 (low-risk group), 35–51 (at-risk group), 52–69 (addicted group), and 70–85 (high-level addicted group) [41]. The internal reliability of the scale is 0.81 (omega coefficient) and 0.80 (Cronbach’s α) [41].

### 2.6. Statistical Analyses

Descriptive statistics were used to summarize participants’ sociodemographic data through frequency tables. The Kolmogorov–Smirnov test was conducted to assess normality, yielding *p*-values < 0.05. Spearman correlation analysis was performed to examine relationships between scale and survey scores. Group differences were analyzed using the Mann–Whitney U test and the Kruskal–Wallis H test. Due to variance heterogeneity and unequal sample sizes, the Games-Howell post hoc test was employed to compare means across multiple groups. The chi-square test or Fisher’s exact test was used to compare the frequencies of genotypes and alleles between different groups. For the meta-analysis of allele frequencies, Cochrane Review Manager (RevMan, v5.3, London, UK) was used. Random-effects models were applied; odds ratios (ORs) with 95% CIs were estimated using the Mantel–Haenszel method, and heterogeneity was assessed with the I2 statistic. A *p*-value < 0.05 was considered statistically significant.

## 3. Results

### 3.1. Association Between Exercise Addiction and Injury Susceptibility

The injury history data revealed that 84% of Turkish volleyball players experienced at least one severe injury, while 26% required surgical intervention for treatment. Additionally, 31.25% of athletes returned to their sport within 1–3 weeks following an injury (Table 2).

The distribution of participants based on their scores from the Exercise Addiction Scale (EAS) is summarized in Table 3. The scoring categories are as follows: 1–17 (normal group), 18–34 (low-risk group), 35–51 (risk group), 52–69 (addicted group), and 70–85 (high-level addicted group). The participants were classified as dependent based on their total EAS score. The Mann–Whitney U test and Kruskal–Wallis test were used to assess differences in sociodemographic characteristics and EAS outcomes. No significant differences were observed in total or subscale EAS scores concerning sociodemographic variables, duration of volleyball participation, familial involvement in sports, surgical history, or dietary supplement use.

Table 4 presents a comparison of overall and subscale EAS scores based on injury severity. The “Delaying of Social Needs and Conflict” subscale score was significantly higher among injured athletes compared to non-injured athletes (Z = −2.099, *p* = 0.036, effect size (r) = −0.30).

Table 5 highlights a statistically significant difference in exercise addiction among athletes who consumed collagen supplements, particularly in the “Tolerance Development and Passion” subscale. Athletes in the no-response group had significantly higher scores on this subscale than those in the yes-response group (Z = −2.561, *p* = 0.01, effect size (r) = −0.036).

### 3.2. Association Between the COL1A1 rs1800012 Polymorphism, Volleyball Player Status, and Injury Susceptibility

The distribution of the *COL1A1* rs1800012 genotypes among the Turkish (*p* = 0.2497, χ^2^ = 1.325) and Russian (*p* = 0.353, χ^2^ = 0.8637) volleyball players was consistent with Hardy–Weinberg equilibrium. In the Turkish cohort, the genotype frequencies were 72% for GG and 28% for GT, with no TT carriers detected. Among the Russian players, the frequencies were 77.4% for GG and 22.6% for GT, also with no TT genotypes. Table 6 presents the allelic frequencies in the Turkish and Russian volleyball players and their respective control groups.

**Figure 1:**
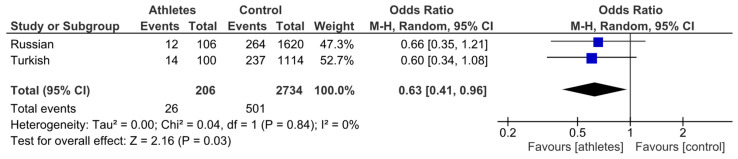
Meta-analysis of association studies examining the *COL1A1* gene and volleyball player status. The under-representation of the *COL1A1* T allele is evident in the volleyball players. The purple squares indicate the proportion of the T allele among volleyball players in each individual study, while the black diamond represents the pooled proportion of the T allele across all volleyball players, along with its 95% confidence interval (CI).

Although the separate analyses showed no statistically significant differences between athletes and controls, the random-effects meta-analysis demonstrated a significantly lower prevalence of the *COL1A1* T allele in volleyball players compared to controls (pooled OR = 0.63, 95% CI: 0.41–0.96, *p* = 0.03), with no evidence of heterogeneity between the Russian and Turkish cohorts (I^2^ = 0%) (Figure 1).

**Figure 2:**
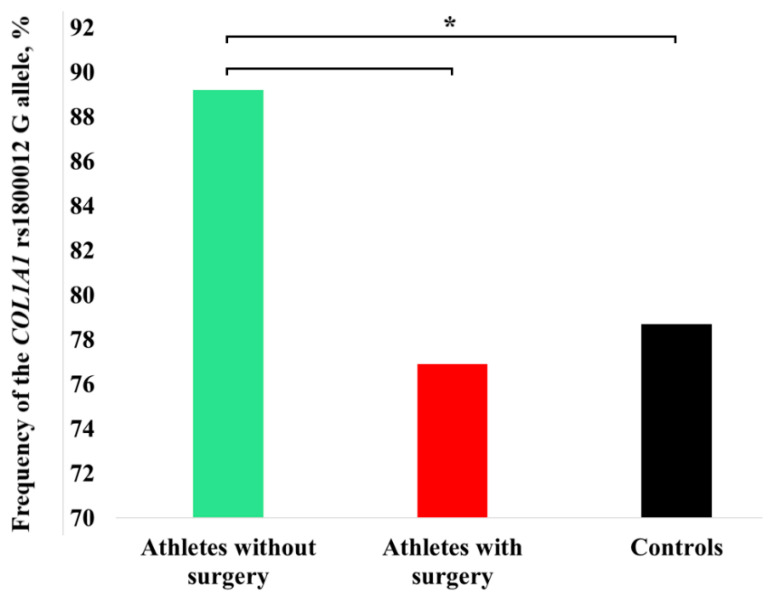
*COL1A1* rs1800012 G allele frequency differences among Turkish athletes who had not undergone surgery (n = 37), athletes who had undergone surgery (n = 13), and controls (n = 557). * *p* = 0.037 (Fisher’s exact test), indicating statistical significance.

No significant differences in EAS scores were found between athletes with different *COL1A1* rs1800012 genotypes (*p* < 0.05). Athletes who had not undergone surgery (n = 37) exhibited a significantly higher frequency of the rs1800012 G allele compared to 557 controls (89.2% Vs. 78.7%, *p* = 0.037; OR = 2.23, 95% CI: 1.1–4.7). However, due to the small sample size, the G allele frequency did not differ significantly between the athletes without and with surgical history (76.9%, *p* = 0.185) (Figure 2).

A statistically significant difference was observed among *COL1A1* genotypes in relation to injury recovery time. Athletes with the GG genotype demonstrated a significantly faster post-injury recovery compared to those with the GT genotype (*p* = 0.009) (Table 7). Furthermore, among the injured athletes, those carrying the GT genotype were significantly more likely to experience prolonged recovery (≥3 months) (57.1%) compared to those with the GG genotype (28.0%, *p* = 0.017).

## 4. Discussion

Our findings indicate that exercise addiction is associated with a higher risk of injury in female volleyball players, and the *COL1A1* rs1800012 T allele is linked to prolonged recovery times. Furthermore, a pooled meta-analysis suggests that the T allele is underrepresented in elite volleyball players compared to the general population, which may indicate a genetic selection effect. However, several important limitations must be considered, including the cross-sectional nature of our data, which precludes causal inferences, and the relatively small sample size of the injury cohort, which limits the statistical power.

Sports injuries arise from the interplay of various factors, including genetic predispositions, environmental influences, and lifestyle choices [9,12,32]. Exercise is categorized as one of 40 potentially addictive activities. Our findings indicated that professional female volleyball players were classified into the exercise addiction group. The sports psychology literature extensively demonstrates that overtraining and addiction can result in psychological stress and physical injury [42]. Measurements utilizing the Exercise Addiction Scale (EAS) indicated that a considerable percentage of participants exhibited elevated addiction scores, which correlated with injury levels. Juwono et al. [15] showed that professional athletes possess a greater susceptibility to exercise addiction compared to non-athletes. These results underscore the importance of meticulously designing and implementing training loads. In contrast to the existing literature, our research aimed to elucidate the multifactorial risk of sports injuries by investigating the interplay between genetic predispositions and psychological factors related to exercise addiction and sports injuries through a comprehensive approach [43,44].

Exercise addiction, characterized by excessive and uncontrolled exercise behaviors, can adversely impact individuals’ physical, psychological, and social well-being by heightening their risk of injury [42,45]. Research indicates that athletes engaged in competitive activities exhibit greater exercise addiction scores compared to those participating in sports for leisure [46,47,48]. All individuals in our study belong to the addicted category, according to the literature. Our results indicate that the “Delay of Individual Social Needs and Conflict” score, a subscale of Exercise Addiction, is higher in those with injuries compared to those without injuries. This outcome indicates that exercise addiction is not solely a physical problem; it also impacts individuals’ social and psychological well-being. The challenges faced by injured athletes in resuming training may compel them to intensify their activity, thereby deferring both social and personal needs.

Numerous studies in the literature have investigated the correlation between exercise addiction and sports injuries. Overtraining and elevated drive can impose psychological and physical stress on athletes, particularly in highly competitive settings. The results of our investigation corroborate the existing literature and indicate that the subdimensions of exercise addiction may be significant for injury risk and management [11,49]. Analysis of the link between exercise addiction and injuries revealed that injured athletes exhibited higher scores on the “Delaying of Personal Social Needs and Conflict” subscale. This suggests that intensive training programs may negatively affect athletes’ recovery and increase the risk of injury when combined with mental fatigue. Our findings align with prior research and suggest that subdimensions of exercise addiction may be associated with injury and its prevention [11,49]. A comprehensive evaluation of athletes’ physical and psychological conditions, early identification and treatment of exercise addiction concerns, and the formulation of preventative strategies are essential for minimizing injury risk and promoting career longevity. The observation that scores on the “Postponement of Individual Social Needs and Conflict” subscale were elevated among injured athletes suggests that these individuals may encounter difficulties in balancing their social and personal lives, perhaps accelerating their risk of injury.

Khoschnau et al. [50] demonstrated that gene polymorphisms may confer protective benefits against sports injuries. Nonetheless, evidence suggests that data from these studies may vary between particular sports and genders. The literature indicates that *COL1A1* gene polymorphism is particularly significant in tendon and ligament structures and may influence the risk of injury to these tissues [51,52]. The predominance of the GG genotype and the lower prevalence of the GT genotype may indicate that these genotypes could have varying impacts on injury duration, injury risk, and the recovery process [19].

Our data suggest that individuals with the GT genotype typically endure prolonged recovery durations compared to those with the GG genotype. A considerable proportion of GT players require three to four months or more for recovery. Moreover, individuals possessing the GT genotype have heightened susceptibility to serious injury. A greater percentage of GT individuals required surgery compared to GG individuals, suggesting a possible trend for extended rehabilitation. The GT genotype is correlated with an increased propensity for serious injuries and extended recovery periods. In a study including 114 participants, Leźnicka et al. [34] found that the *COL1A1* rs1800012 GT genotype correlated with a 2.2-fold increased risk of injuries. These findings align with prior studies suggesting that the T allele may be a risk factor for soft tissue injuries and prolonged healing periods [35]. Athletes possessing the GT genotype may exhibit increased susceptibility to injuries due to a collagen structure with diminished resilience. The underlying mechanism may involve the *COL1A1* rs1800012 G>T substitution, which reduces type I collagen expression and leads to weaker fibers that are essential for tendon and ligament strength, as supported by GTEx data showing lowest expression in TT carriers.

The beneficial effects of collagen supplementation on tendon and ligament health are often highlighted in the literature [53]. However, collagen supplementation did not have a significant effect on injury rates in our study. To clarify this relationship more comprehensively, future studies should include a larger number of athletes from various sports disciplines. An intriguing exploratory observation from our study was the association between collagen supplementation and lower scores on the ‘Tolerance Development and Passion’ subscale of the EAS. This finding should be interpreted with caution. It may suggest that athletes who consciously use collagen supplements adopt a more measured approach to training, potentially as part of a broader injury prevention mindset, which is reflected in a different psychological profile. Alternatively, it could indicate that supplements mitigate physical sensations that drive the need to increase training volume. However, this is a preliminary correlation from a cross-sectional analysis and does not imply causation. The direction of this relationship is unclear—supplementation could influence psychology or a certain psychological profile could influence the decision to supplement. Furthermore, we found no significant association between collagen use and a reduction in severe injuries or surgery rates in this small cohort. This observation remains hypothesis-generating and warrants focused investigation in future longitudinal or interventional studies that control for dosage, duration, and underlying motivations for use.

This study has several limitations that should be acknowledged. First, the cross-sectional design does not allow for causal interpretations, and self-reported data on collagen use and exercise behaviors may be subject to recall or response bias. Second, although the Exercise Addiction Scale used showed acceptable internal reliability, further validation across broader non-Turkish populations is needed. Third, the observed associations, particularly those related to collagen supplementation and genotype differences, should be interpreted with caution and warrant further investigation in larger, longitudinal studies. Replication of these findings in other ethnicities is also required to confirm their generalizability, as recommended in sports genetics research [52,54,55]. Fourth, the small sample size of the primary injury cohort (n = 50) reduces the statistical power for the analyses of injury, genetics, and exercise addiction, increasing the risk of Type II errors and limiting the generalizability of these findings. The absence of injury data for the Russian cohort further prevented cross-validation of the injury-genetics associations. Additionally, the lack of detailed information on training load and physical activity levels in the control populations weakens the behavioral context of the genetic case–control comparisons. Fifth, DNA sources and genotyping platforms differed between the Turkish and Russian cohorts (oral swabs and real-time PCR versus leukocytes and microarray/sequencing). While the genotype for a given SNP is consistent across an individual’s somatic cells and both methods are highly accurate, this technical heterogeneity could theoretically contribute to batch effects. Finally, the analysis was restricted to a single genetic variant, whereas numerous other variants (such as such *COL5A1*, *ACTN3*, *MMP3*, *VEGFA*, etc.) are known to influence injury risk [20,27,28,29,30,31,34]. This monogenic approach is a significant limitation as it cannot capture the complex, polygenic nature of sports injury susceptibility. Future research should move toward the development of polygenic risk scores (PRSs), an approach that has been successfully attempted in several recent studies to aggregate the small effects of many variants and provide a more comprehensive assessment of genetic risk [56,57,58,59].

## 5. Conclusions

In summary, this study provides evidence that both genetic predisposition, indicated by the *COL1A1* rs1800012 polymorphism, and psychological factors, such as exercise addiction, are associated with injury susceptibility in elite female volleyball players. The underrepresentation of the T allele in the athletic cohort suggests a potential genetic selection pressure in this high-performance environment, while the link between collagen supplementation and exercise psychology presents an intriguing, preliminary finding for future inquiry. It is crucial to interpret these results as exploratory associations, not causal relationships, due to the cross-sectional design and sample size limitations. Nonetheless, our findings contribute to the understanding of the biopsychogenetic mechanisms that underpin athlete resilience. This work underscores the necessity of a holistic approach in sports medicine and provides a foundational rationale for future longitudinal studies with larger cohorts. Such research is essential to confirm these findings and translate them into personalized strategies for injury prevention and rehabilitation, ultimately enhancing athlete health and performance.

## Figures and Tables

**Table 1:** Characteristics of Turkish participants.

Variable	Value	
Age		
Mean ± SD	26.2 ± 4.65	
Median (min–max)	25.5 (19–38)	
Height (cm)		
Mean ± SD	178.9 ± 8.54	
Median (min–max)	180.0 (158–199)	
Weight (kg)		
Mean ± SD	67.9 ± 7.6	
Median (min–max)	68.5 (50–83)	
Position	*n*	%
Libero	12	24.0
Middle player	15	30.0
Setter	9	18.0
Spiker	14	28.0
Duration of play		
4–7 years	3	6.0
8–10 years	6	12.0
≥11 years	41	82.0
Collagen supplementation		
Yes	37	74.0
No	13	26.0

**Table 2:** Injury history of Turkish volleyball players.

Variable		*n*	%
Severe Injury	1–5	42	84.0
	None	8	16.0
Surgery	Yes	13	26.0
	No	37	74.0
Return to sport following injury	1–3 weeks	15	31.25
	1–2 months	9	18.75
	3–4 months	3	6.25
	≥5 months	12	25.00
	No injuries	9	18.75

**Table 3:** Distribution of participants based on Exercise Addiction Scale scores.

EAS Parameter	Min	Max	Mean ± SD
Total EAS score	40.0	71.0	56.12 ± 7.49
EAS–Extreme focus and emotional transformation	15.0	34.0	26.02 ± 4.04
EAS–Delaying of personal social needs and conflict	11.0	28.0	18.42 ± 3.65
EAS–Development of tolerance and passion	6.0	18.0	11.68 ± 2.53

**Table 4:** Comparison of EAS overall and subscale scores based on severe injury occurrence.

Variable	Experienced Severe Injury	*n*	Mean ± SD	Z	*p* and *r*
EAS (Total)	1–5	42	56.76 ± 7.63	−1.524	0.128; −0.22
	None	8	52.75 ± 5.99		
EAS–Extreme focus and emotional transformation	1–5	42	25.95 ± 4.29	−0.160	0.873; −0.02
	None	8	26.38 ± 2.56		
EAS–Delaying of personal social needs and conflict	1–5	42	18.90 ± 3.61	−2.099	0.036 *; −0.30
	None	8	15.88 ± 2.80		
EAS–Development of tolerance and passion	1–5	42	11.90 ± 2.53	−1.378	0.168; −0.19
	None	8	10.50 ± 2.33		

* *p* < 0.05 (Mann–Whitney U Test), r = effect size (rank biserial correlation).

**Table 5:** Comparison of EAS subscale scores based on collagen supplementation.

Variable	Collagen Supplementation	*n*	Mean ± SD	Z	*p* and *r*
EAS (Total)	Yes	37	55.70 ± 7.15	−0.875	0.382; −0.12
	No	13	57.31 ± 8.57		
EAS–Extreme focus and emotional transformation	Yes	37	25.59 ± 3.83	−1.515	0.130; −0.21
	No	13	27.23 ± 4.53		
EAS–Delaying of personal social needs and conflict	Yes	37	18.92 ± 3.74	−1.61	0.107; −0.23
	No	13	17.0 ± 3.08		
EAS–Development of tolerance and passion	Yes	37	11.19 ± 2.26	−2.561	0.01 *; −0.36
	No	13	13.08 ± 2.81		

* *p* < 0.05 (Mann–Whitney U Test), r = effect size (rank biserial correlation).

**Table 6:** Allelic frequencies of the *COL1A1* rs1800012 polymorphism in Turkish and Russian athletes and controls.

Group	n	T Allele	G Allele	T Allele Frequency, %	G Allele Frequency, %
Turkish athletes	50	14	86	14.0	86.0
Turkish controls	557	237	877	21.3	78.7
Russian athletes	53	12	94	11.3	88.7
Russian controls	810	264	1356	16.3	83.7

**Table 7:** Association between *COL1A1* rs1800012 genotypes and recovery time following injury.

Return to Sport Following Injury	*COL1A1* rs1800012 Genotypes		*p*
	GG	GT	
1–3 weeks	10	5	0.009 *
1–2 months	8	1	
3–4 months	0	3	
≥5 months	7	5	
No injuries	9	0	

* *p* < 0.05 (chi-square test), indicating a statistically significant relationship between *COL1A1* rs1800012 genotypes and return to sport after injury.

## Data Availability

The original contributions presented in this study are included in the article. Further inquiries can be directed to the corresponding authors.

## Abbreviations

The following abbreviations are used in this manuscript:

ACL	Anterior cruciate ligament
COL1A1	Collagen Type I Alpha 1 Chain
DNA	Deoxyribonucleic acid
EAS	Exercise Addiction Scale
PCR	Polymerase chain reaction
SNP	Single-nucleotide polymorphism
